# In Vitro Evaluation of Aryl Hydrocarbon Receptor Involvement in Feline Coronavirus Infection

**DOI:** 10.3390/v17020227

**Published:** 2025-02-06

**Authors:** Luca Del Sorbo, Rosa Giugliano, Claudia Cerracchio, Valentina Iovane, Maria Michela Salvatore, Francesco Serra, Maria Grazia Amoroso, Francesco Pellegrini, Martina Levante, Paolo Capozza, Georgia Diakoudi, Massimiliano Galdiero, Giovanna Fusco, Annamaria Pratelli, Anna Andolfi, Filomena Fiorito

**Affiliations:** 1 Department of Veterinary Medicine and Animal Production, University of Naples Federico II, 80137 Naples, Italy; luca.delsorbo2@studenti.unina.it (L.D.S.); rosa.giugliano@unicampania.it (R.G.); claudia.cerracchio@unina.it (C.C.); 2 Department of Experimental Medicine, University of Campania Luigi Vanvitelli, 80138 Naples, Italy; massimiliano.galdiero@unicampania.it; 3 Department of Agricultural Sciences, University of Naples Federico II, 80055 Portici, Italy; valentina.iovane@unina.it (V.I.); andolfi@unina.it (A.A.); 4 Department of Chemical Sciences, University of Naples Federico II, 80126 Naples, Italy; 5 Istituto Zooprofilattico del Mezzogiorno, 80055 Portici, Italy; francesco.serra@izsmportici.it (F.S.); mariagrazia.amoroso@izsmportici.it (M.G.A.); martina.levante@izsmportici.it (M.L.); 6 Department of Veterinary Medicine, University of Bari, 70010 Valenzan, Italy; francesco.pellegrini@uniba.it (F.P.); paolo.capozza@uniba.it (P.C.); georgia.diakoudi@uniba.it (G.D.); 7 BAT Center-Interuniversity Center for Studies on Bioinspired Agro-Environmental Technology, University of Naples Federico II, 80055 Portici, Italy

**Keywords:** FCoV, AhR signaling, CH223191, lysosomes, antiviral activity, in vitro system

## Abstract

Feline coronavirus (FCoV) is an alphacoronavirus (αCoV) that causes moderate or chronic asymptomatic infection in cats. However, in a single infected cat, FCoV can modify its cellular tropism by acquiring the ability to infect macrophages, resulting in the development of feline infectious peritonitis (FIP). In this context, to restrain the impact of FCoV infection, scientific research has focused attention on the development of antiviral therapies involving novel mechanisms of action. Recent studies have demonstrated that aryl hydrocarbon receptor (AhR) signaling regulates the host response to different human and animal CoVs. Hence, the mechanism of action of AhR was evaluated upon FCoV infection in Crandell Feline Kidney (CRFK) and in canine fibrosarcoma (A72) cells. Following infection with feline enteric CoV (FECV), strain “München”, a significant activation of AhR and of its target CYP1A1, was observed. The selective AhR antagonist CH223191 provoked a reduction in FCoV replication and in the levels of viral nucleocapsid protein (NP). Furthermore, the effect of the AhR inhibitor on the acidity of lysosomes in infected cells was observed. Our findings indicate that FCoV acts on viral replication that upregulates AhR. CH223191 repressed virus yield through the inhibition of AhR. In this respect, for counteracting FCoV, AhR represents a new target useful for identifying antiviral drugs. Moreover, in the presence of CH223191, the alkalinization of lysosomes in FCoV-infected CRFK cells was detected, outlining their involvement in antiviral activity.

## 1. Introduction

Coronaviruses (CoVs) are enveloped positive-sense, single-stranded RNA viruses that can infect several hosts, including mammals and birds. CoVs can cross interspecies barriers and undergo genetic evolution. From their natural reservoirs, such as migratory birds or bats, CoVs can accumulate mutations in intermediate hosts, giving rise to novel strains, sometimes presenting severe changes in virulence and tissue tropism [1,2,3,4,5,6]. Over the past two decades, animal CoVs have jumped to humans at least three times, and in 2019, they caused one of the most serious pandemics in modern human history (COVID-19). In addition, the recent detection of CCoV-HuPn-2018 [7] and HuCCoV-Z19Haiti [8], novel canine–feline recombinant alphacoronaviruses (αCoVs) isolated from human patients, once again highlights the interspecies transmission of CoVs. 

Feline coronavirus (FCoV), an αCoV, belongs to the *Orthocoronavirinae* subfamily, *Coronaviridae* family, *Nidovirales* order, and includes two biotypes: feline enteric coronavirus (FECV), which generally induces moderate enteritis or chronic asymptomatic infection in cats, and feline infectious peritonitis virus (FIPV), which, in a low percentage of infected cats, can develop severe feline infectious peritonitis (FIP) [1,9,10]. 

FCoV is closely correlated to both animal and human CoVs, such as canine coronavirus (CCoV), transmissible gastroenteritis virus (TGEV) of pigs, and CCoV-HuPn-2018 in humans [7]. Two serotypes of FCoV exist, FCoV-I and FCoV-II, the latter being the evolutionary result of a recombination event between FCoV and CCoV affecting the S and M genes [3,11,12]. Based on their pathobiology, FCoVs are classified into two biotypes, FECV, that typically induces mild or subclinical enteritis and affecting cats in multi-cat households (up to 90%) [13], and FIPV, a spontaneous mutation of FECV responsible for FIP, a fatal viral disease, affecting wild and domestic felines globally. The mutations developed in the individual cat result in a change in cell tropism from enterocytes to monocytes, resulting in the development of high pathogenic FIP [1,10]. FIPV generally affects purebred cats younger than two years of age (4 to 16 months) [10,14]. FIPV can cause severe inflammatory damage to different organs, including the lungs, lymphoid tissues, heart, eyes, liver, and brain [1,10]. 

The emergence of a new, highly pathogenic FCoV-CCoV recombinant responsible for a rapidly spreading outbreak of FIP in cats of all ages in Cyprus was recently described [15,16]. Interestingly, it was hypothesized that the new strain is the result of a deletion and amino acid changes in the spike protein, and studies showed a very high sequence identity (97%) with the pantropic CCoV CB/05 [17,18,19,20,21]. These features may mainly modify the receptor binding domain, suggesting changes in receptor binding and cell tropism compared to other FCoVs-II [21].

The aryl hydrocarbon receptor (AhR) is a transcription factor stimulated by various substrates, both endogenous, such as bilirubin and biliverdin, and exogenous, including environmental contaminants (dioxin) and microbial metabolites. Thus, AhR is implicated in various physiological functions, involving both embryonic and adult tissue development, chemical and microbial defense, and energy metabolism. In the canonical AhR pathway, after ligand binding, the AhR–ligand complex translocates into the nucleus, where it acts with specific DNA sequences, to control the gene expression that encodes cytochrome P450 enzymes (such as CYP1A1, CYP1B1, and CYP2A1), inducing cytokine release and the regulation of immune responses [22,23]. AhR activation also stimulates a negative-feedback loop that acts on the expression of the AhR repressor (AhRR), replacing AhR, which is then transferred to the cytosol where the 26S proteasome biodegrades it [22]. During infection, AhR interferes with natural protective immune responses to various microorganisms [22,23,24,25,26,27]. The study of the virus-cell–host interaction allowed for the identification of AhR as a pro-viral host factor of both αCoVs and βCoVs. For example, following CoVs infection with murine coronavirus (MCoV), CCoV type II, Middle East respiratory syndrome coronavirus (MERS-CoV), human coronavirus (HCoV) 229E, severe acute respiratory syndrome CoV type 1 (SARS-CoV-1), and SARS-CoV-2 [27,28,29,30,31,32,33], AhR activation was found. During MCoV infection, the inhibition of AhR with the selective AhR antagonist CH223191 induces a decline in the expression of cytokines interleukin (IL)-1b and (IL)-10, as well as an enhancement in the expression of the tumor necrosis factor, while converse modulation is caused by a specific agonist (dioxin) [22]. Furthermore, CH223191, together with the small-molecule AhR-inhibitor 3′,4′-dimethoxy-α-naphthoflavone (DiMNF), inhibits the replication of SARS-CoV-2, HCoV-229E, and CCoV [29,30,31,32]. In addition, the involvement of AhR during CoVs infection [34,35,36,37] was demonstrated, implicating AhR as a possible druggable target to counteract CoVs infection.

In this study, it was demonstrated that FCoV infection activated AhR, which is expressed in both Crandell Feline Kidney (CRFK) and canine fibrosarcoma (A72) cells. The pharmacological action of CH223191, downregulating AhR, provoked inhibition in FCoV replication. Hence, AhR deserves attention in the design of FCoV antiviral strategies. Moreover, considering that lysosome alkalinization occurred during CoVs infection [38], the potential effect of the AhR inhibitor CH229131 on lysosomes pH during FCoV infection was also evaluated in vitro in CRFK cells. Taken together, our results extend the knowledge on host–pathogen dynamics. 

## 2. Materials and Methods

### 2.1. Ethical Approval

This study did not require ethical approval.

### 2.2. Cell Cultures and Virus Infection

CRFK and A72 cells were cultured in Dulbecco’s modified Eagle’s minimal essential medium (DMEM) supplemented with 10% fetal bovine serum (FBS) and incubated at 37 °C and 5% CO_2_ [36,39]. FCoV type I, biotype FECV (isolate “München”), kindly provided by the Friedrich Loeffler Institute (FLI, Insel Riems, Germany; viral registration number RVB-1259) was employed throughout this study. CRFK cells were used for virus stock growth and virus titration [40,41,42,43].

The synthetic and specific AhR competitive antagonist, 2-Methyl-2H-pyrazole-3-carboxylic acid (2-methyl-4-*O*-tolylazo-phenyl)-amide (CH223191) (Sigma-Aldrich, St. Louis, MI, USA), [22,44], was solubilized in dimethyl sulfoxide (DMSO) (Sigma-Aldrich, St. Louis, MI, USA) in order to obtain 2, 5, 10, and 20 µm [29,30]. Monolayers of CRFK and A72 [45] cells were pretreated for 1 h at 37 °C with DMEM supplemented with 10% FBS containing different concentrations of CH223191 (2, 5, 10, and 20 µm). Then, the cell lines were either infected or not infected with a tissue culture of infectious dose 50 (TCID_50_) of 1 × 10^7.33^/mL of FCoV isolate “München”, at a multiplicity of infection (MOI) of 0.05 or 0.5 to obtain four groups/cell line: (a) untreated uninfected cells; (b) untreated infected cells; (c) CH223191-treated uninfected cells; (d) CH223191-treated infected cells. After FCoV adsorption for 1 h at 37 °C, cells were then incubated. At 24 h post-infection (p.i.), cells were processed. The virus was in the culture medium during the experiment.

### 2.3. Cell Viability 

Cell viability was assessed using Trypan Blue (TB) (Sigma-Aldrich) exclusion test. Briefly, monolayers of CRFK and A72 cells, pretreated or not pretreated with CH223191 at different concentrations (2, 5, 10, and 20 µm) were either infected or not infected with FCoV at a MOI of 0.05 for 24 h, collecting cells through trypsinization and mixing a volume of the cellular suspension and an equivalent quantity of 0.2% Trypan Blue (Sigma) in 1×phosphate-buffered saline (PBS) for 10 min. Cells were counted through TC20 automated cell counter (Bio-Rad). The number of viable cells in the total number of cells was determined as a percentage, and the results were reported as the mean ± S.D. of three independent experiments performed twice. The 50% cytotoxicity concentrations (CC_50_) of CH223191_,_ which is the cytotoxic concentration of the inhibitor required to reduce cell viability by 50%, were identified, and a dose–response curve was obtained in analyzed cells. Additionally, the 50% inhibitory concentration (IC_50_) was evaluated by Calculator|AAT Bioquest (https://www.aatbio.com/tools/ic50-calculator, accessed on 2 December 2024). Furthermore, cell viability was determined using Trypan Blue in cells as previously reported [46,47]. The results were shown as the mean ± SD of four independent experiments in duplicate.

### 2.4. Cell Proliferation 

To analyze cell proliferation, Cell Proliferation Kit I 3-(4,5-dimethyl-2-thiazolyl)-2,5-diphenyl-2H-tetrazolium bromide (MTT) (Roche) assay was used, as previously described [48]. Briefly, CRFK and A72 cells cultured in 96-well plates, pretreated or not pretreated with CH223191 at different doses (2, 5, 10, and 20 µm), were infected with FCoV, at MOI of 0.05, and incubated. At 24 h p.i., MTT assay was carried out, measuring the absorbance at 540 nm (A_540_). The results were expressed as the mean S.D. of four independent experiments performed twice.

### 2.5. Examination of Cell Morphology

In a 24-well plate, cell monolayers of CRFK (1.0 × 10^5^/well), pretreated or not pretreated with CH223191, were either infected or not infected with FCoV at MOI 0.5 for Giemsa staining and at MOI 0.05 for AO/PI, and incubated for 24 h. Monolayers of A72 cells (5.0 × 10^4^/well), pretreated or not pretreated with CH223191, were either infected or not infected with FCoV at MOI 0.5 for both Giemsa and AO/PI staining, and incubated for 24 h. Then, cells were washed twice with PBS and stained with Giemsa and acridine orange/propidium iodide (AO/PI) to analyze cell morphology [36,49]. To detect the cell death features, previously described criteria were used [49,50,51]. 

Giemsa staining was performed by fixing cells with 95% ethanol, then drained and dried. Subsequently, cells were stained with a 5% Giemsa solution (Merck, Darmstadt, Germany), and, after 30 min, the cells were rinsed with tap water and H_2_O. Light microscopy analyses were performed by a ZOE Cell Imager (Bio-Rad Laboratories, Hercules, CA, USA).

To detect both viable and dead cells, the cells were stained with acridine orange/propidium iodide (AO/PI) (1 µg/mL each) for 15 min at 37 °C, washed with PBS, and observed by fluorescence microscopy ZOE Cell Imager (Bio-Rad Laboratories, Hercules, CA, USA). Fluorescent substances, used in combination, permit the recognition of undamaged or compromised membranes [50]. Due to the membrane permeability of AO, it binds nucleic acids developing green fluorescence. Due to the impermeability of the intact cell membrane to PI, the membrane of dead and dying cells can only be crossed by PI. So, it can intercalate nucleic acids, generating a complex with bright red fluorescence. The fluorescence signals from microscopy images were quantified by ImageJ (National Institutes of Health) software (Java 1.8.0_345).

### 2.6. Immunofluorescence Staining

In a 96-well plate, CRFK (2.0 × 10^4^/well) and A72 (1.0 × 10^4^/well) cell monolayers, pretreated or not pretreated with CH223191, were either infected or not infected with FCoV, at MOI 0.05. Immunofluorescence staining was carried out at 24 h p.i. [36,52], by fixation in 4% paraformaldehyde for 20 min. Then, cells were permeabilized with 0.1% Triton X100 for 10 min. Blocking was achieved by 2% BSA (Sigma-Aldrich, St. Louis, MI, USA) for 45 min, followed by incubation at 4 °C, overnight with primary antibodies, diluted in 5% bovine serum albumin-1x Tris-Buffered Saline, 0.1% Tween® 20 Detergent: (i) anti-aryl hydrocarbon receptor (AhR) (Sigma-Aldrich, St. Louis, MI, USA) (1:250), (ii) monoclonal mouse anti-FCoV N protein (MCA2194, Bio-Rad) (1:400); (iii) monoclonal mouse anti-CYP1A1 (A-9) (sc-393979, Santa Cruz Biotechnology, Inc., Dallas, TX, USA) (1:250); (iv)Texas Red goat anti-rabbit (Thermo Fisher Scientific, Waltham, MA, USA) (1:500), (v) Alexa Fluor 488 goat anti-mouse (Thermo Fisher Scientific) (1:500). Microscopy and photography were both evaluated via ZOE Fluorescent Cell Imager (Bio-Rad Laboratories). The fluorescence signals from microscopy images were quantified by ImageJ (National Institutes of Health) software. The fluorescence intensity was determined and plotted versus the control group (DMSO).

### 2.7. Virus Production

CRFK and A72 monolayers in a 24-well plate, pretreated or not pretreated with CH223191, were either infected or not infected with FCoV, at an MOI of 0.5, incubated at 37 °C, and processed after 24 h of infection by real-time PCR for FCoV in triplicate and repeated twice.

CRFK cell extracts were obtained after three cycles of freezing and thawing, and then collected, aliquoted, and stored at −80 °C. The virus titration was performed using the TCID_50_ method in a 96-well plate, according to Reed and Muench (1938), as previously reported [53]. The TCID_50_ method could not be used because the CPE induced by FCoV in A72 is generally poorly evident [46]. After seeding the cells in a 96-well plate, the cytopathic effect (CPE) was assayed at 24 h p.i., after washing, fixing with methanol, and staining cells with crystal violet (0.1% *w/v*) (Sigma-Aldrich) [54,55].

### 2.8. RT-qPCR for FCoV in CRFK and A72 Cells

RT-qPCR was employed for the detection of FCoV RNA. The extraction of nucleic acids was carried out on cell supernatant (200 µL) by the commercial QIAsymphony automated extraction system (Qiagen GmbH, Hilden, Germany) with the DSP Virus/Pathogen Mini kit (Qiagen GmbH, Hilden, Germany) according to the manufacturer’s instructions. Subsequently, nucleic acids were eluted in 60 µL of the elution buffer. Before sample extraction, murine norovirus (MuNoV) [56] was included to each sample, useful for determining viral recovery and for the detection of PCR inhibitors (external process control, EPC). The analysis of results was performed considering that when the threshold cycle (Ct) of the EPC in the eluted sample is similar to that of the EPC in the NPC, the undiluted sample is analyzed. Meanwhile, when the difference between the two Cts is at least 3 or a multiple of 3, the sample diluted to 1:10 or more was used to perform the analysis [57] using a QuantStudio 5 Real-Time PCR thermal cycler (Thermo Fisher Scientific, Waltham, MA, USA) in a total volume of 25 µL containing 5 µL of nucleic acids extract, 12.5 µL of AGPATH reaction kit with 1 µL of reverse transcriptase enzyme (Thermo Fisher Scientific, Waltham, MA, USA), 1 µL (6.25 µm) of primer forward FCoV-For (5′-AGCAACTACTGCCACRGGAT-3′), 1 µL (6.25 µm) of primer reverse FCoV-Rev (5′-GGAAGGTTCATCTCCCCAGT-3′), and 1 µL (5 µm) of probe FCoV-P (5′-FAM-AATGGCCACACAGGGACAACGC-MGB-3′). After reverse transcription for 30 min at 48 °C, thermal cycling consisted of the activation of iTaq DNA polymerase at 95 °C for 15 min, and 45 cycles of denaturation at 95 °C for 15 s and annealing extension at 60 °C for 60 s [58]. Quantification was performed using a standard curve, for each cell line, obtained by amplifying serial dilutions of the quantified extracted virus (from 1.0 × 10^12^ to 1.0 × 10^6^ TCID_50_/mL) [46]. The graphical representation was constructed by plotting serial dilutions of the known amount of virus (expressed in LogTCID_50_/mL) versus Ct number [46].

### 2.9. Viral Gene Expression for Viral NP and Cellular CYP1A1 Gene Expression in A72 Cells

Real-time PCR was performed to quantify the relative expression levels of the mRNA coding for the viral NP and cellular CYP1A1 in FCoV-infected A72 cells pretreated or not pretreated with the inhibitor CH223191. A72 cells were pretreated or not pretreated with the AhR inhibitor and infected with FCoV at MOI of 0.05 and 24 h later, and then total RNA was extracted with TRIzol^®^ reagent (Thermo Fisher, Waltham, MA, USA). The 5x retrotranscription of 1 μg of RNA into cDNA was performed by all-In-One RT MasterMix (Applied Biological Materials, Richmond, BC, Canada). Then, real-time PCR was performed in duplicate using the Insta Q96-6.0 thermocycler (HiMedia, Modautal, Germany). Briefly, 2 µL of cDNA was amplified in a 20 µL of reaction using BlasTaq 2qPCR mastermix (Applied Biological Materials, Richmond, BC, Canada) and 0.1 µm of primers reported above. The relative target threshold cycle (Ct) values of the interest gene NP, forward (5′-AGCAACTACTGCCACRGGAT-3′) and reverse 5′-GGAAGGTTCATCTCCCCAGT-3′), were normalized with the housekeeping gene, the glyceraldehyde 3-phosphate dehydrogenase (GAPDH), forward (5′-CGGAGTCAACGGATTTGGTCGTAT-3′), and reverse (5-AGCTTCTCCATGGTGGTGAAGAC-3′). The primer sequences used for the CYP1A1 gene were forward (5′-TTTGGAGCTGGGTTTGACAC-3′) and reverse (5-CTGCCAATCACTGTGTCTA-3′). The mRNA levels of cells were expressed using the 2^−ΔΔCt^ method [59]. The calculation of the fold induction of the FCoV gene encoding NP and CYP1A1 proteins was performed in A72-infected cells pretreated with the CH223191 inhibitor compared to infected cells pretreated only with DMSO. We applied the 2^−ΔΔCt^ method, calculating the difference between the Ct values (Δ Ct) of the interest gene and the housekeeping gene (GAPDH) for each sample from which the fold induction was obtained. 

### 2.10. LysoRed Staining

Cell monolayers, infected with FCoV at MOI of 0.5, pretreated or not pretreated with CH223191, were incubated for 24 h. Then, CytoPainter LysoRed Indicator Reagent (Abcam) was used to stain cells, according to the user manual. After washing, cells were analyzed with a fluorescence microscope [60]. Original images used for the quantification of fluorescence are reported as the Supplementary Materials.

### 2.11. Statistical Analysis 

The results are described as mean ± S.D. One-way ANOVA with Tukey’s post-test and by Student’s *t* test was calculated by GraphPad InStat Version 3.00 for Windows 95 (GraphPad Software, San Diego, CA, USA). *p* < 0.05 was considered statistically significant.

## 3. Results

### 3.1. CH223191 Increases Cell Viability During FCoV Infection

**Figure 1:**
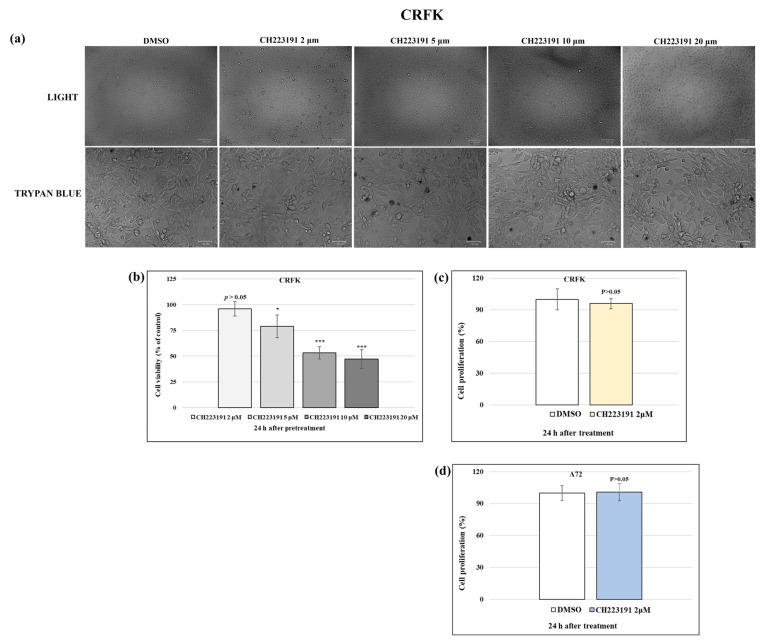
Identification of CC_50_ of CH223191 and generation of dose–response curve in CRFK cells. CRFK cells treated with DMSO or with CH223191 (2, 5, 10, and 20 μM) for 24 h. (**a**) Monolayers of CRFK were either treated or not treated with TB, while cells were attached to wells. Scale bar: 50 µm and 100 µm. (**b**) Dose–response curve of CRFK cells pretreated with CH223191 at different concentrations (2, 5, 10, and 20 μM); cell viability was determined by TB staining and scored cells by automated cell counter. (**c,d**) Dose–response curve of CRFK and A72 cells pretreated with CH223191 (2 μM) for 24 h; cell proliferation was assessed by MTT test. Significant differences between DMSO and CH223191-treated cells are indicated by probability *p*. * *p* < 0.05 and *** *p* < 0.001. Results show the mean ± S.D. of four independent experiments in duplicate.

The impact of CH223191 on FCoV infection was evaluated by analyzing cell viability with TB and cell proliferation with the MTT assay. The effects of CH223191 (2, 5, 10, and 20 μM) on uninfected CRFK and A72 cells were determined 24 h after treatment. The CC_50_ of CH223191 was found, and a dose–response curve was developed in CRFK cells (Figure 1). Cell viability (% control) was detected by TB after 24 h of treatment with a CC_50_ of 5.9 μM CH223191 in CRFK (Figure 1) and 9.6 μM in A72, as reported by us [30]. CH223191 at 2 µm in CRFK cells (Figure 1), as well as in A72 cells [30], provoked no significant differences in cell viability (*p* > 0.05). Therefore, 2 µm of AhR inhibitor, confirmed as biocompatible and non-cytotoxic on CRFK and A72 cells, was used for the experimental design. Indeed, after 24 h of treatment, 2 µm of CH223191 did not generate significant (*p* > 0.05) alterations in the activity of mitochondrial dehydrogenase both in CRFK and in A72 cells compared to the control groups (Figure 1c,d).

**Figure 2:**
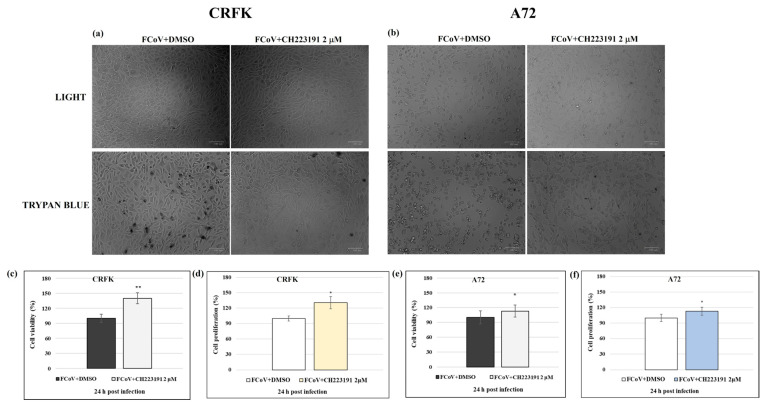
The AhR inhibitor CH223191 determines an increase in cell viability during FCoV infection. CRFK and A72 cells pretreated or not pretreated with CH223191 at 2 µm and infected with FCoV for 24 h. (**a**,**b**) Monolayers of CRFK and A72 cells were unstained or stained with TB and observed with a light microscope, while cells were attached to wells. Scale bar: 100 µm. (**c**,**e**) Dose–response curve of CRFK and A72 cells pretreated with CH223191 at 2 μM. After 24 h of infection, cell viability was determined by TB staining, and cells were scored with an automated cell counter. (**d**,**f**) Dose–response curve of CRFK and A72 cells pretreated with CH223191 at 2 μM. At 24 p.i., cell proliferation was assessed by MTT assay. Significant differences between FCoV + DMSO and FCoV + CH223191-treated cells are indicated by probability *p*. * *p* < 0.05 and **p < 0.01. Results show the mean ± S.D. of four independent experiments in duplicate.

Subsequently, to assess the effect of inhibitor during infection, CRFK and A72 monolayers were pretreated or not pretreated with CH223191 at 2 μM and infected with FCoV at MOI of 0.05 for 24 h. After that, cell viability and proliferation were evaluated. Following FCoV infection, the presence of CH223191 (2 µm) was able to induce a significant increase in cell viability of CRFK (*p* < 0.01) and A72 (*p* < 0.05) cells, as shown in Figure 2. At the same concentration (2 µm), during infection, the inhibitor also intensified CRFK and A72 cell proliferation (*p* < 0.05) (Figure 2).

These data suggested that 2 µm of CH223191 is the optimal non-cytotoxic concentration to use throughout this study, being able to significantly increase cell viability after 24 h from FCoV infection.

### 3.2. CH223191 Reduces Features of Cell Death Morphology During FCoV Infection in CRFK and A72 Cells

**Figure 3:**
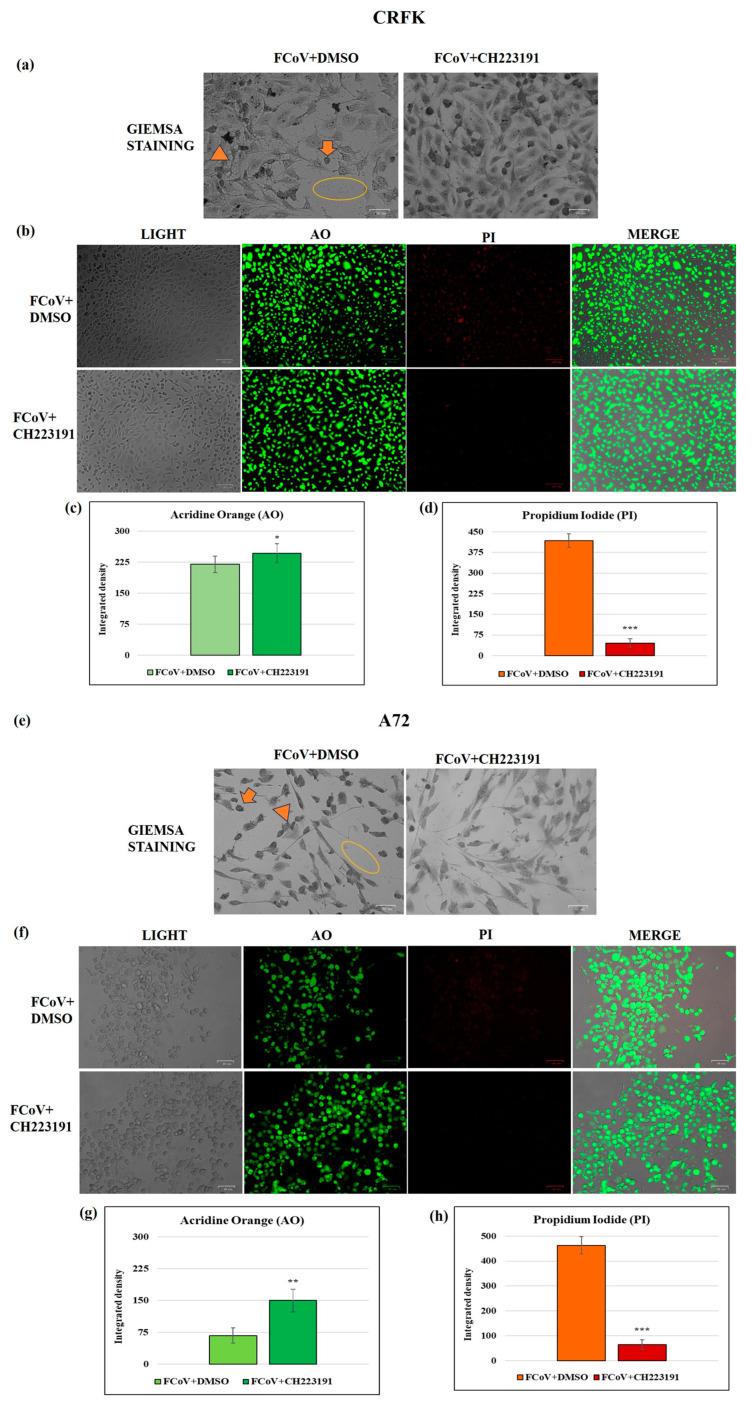
The AhR inhibitor CH223191 diminishes marks of cell death in morphology during FCoV infection in both cell lines. Cells pretreated or not pretreated with CH223191 were infected with FCoV. (**a**–**e**) At 24 h p.i., stained cells by Giemsa were observed with a light microscope. Morphological features of cell death, such as cellular shrinkage (arrowhead), pyknosis (arrow), and detachment of cells from the culture plate (circle), were mostly weakened in CH213191-treated groups in both infected cell lines. (**b**–**f**) In AO/PI panels, PI fluorescent cells, indicating dead and dying cells, were detected in most FCoV-infected CRFK and A72 cells compared to infected cells pretreated with CH213191. Scale bar: 49, 50, and 100 µm. (**c**–**g**) In both cell lines, integrated density quantification showed that the CH223191 treatment provoked an increase in green fluorescence (AO) and (**d**–**h**) a decrease in PI fluorescence of infected cells compared to FCoV untreated groups. Significant differences between FCoV + DMSO- and FCoV + CH223191-treated cells are indicated by probability *p*. * *p* < 0.05, ** *p* < 0.01, and *** *p* < 0.001. The results of one experiment representative of three independent experiments were reported.

In CRFK cells, the pretreatment with 2 μM of the AhR inhibitor, the lowest concentration tested, induced no morphological variations when the uninfected CRFK cell groups were compared to the control; similar results were observed in A72 cells [31] (Figure 1). In contrast, an increase in intercellular spaces due to detachment from the culture plate was found in untreated infected CRFK and A72 cells (Figure 2). Morphological signs of apoptosis were observed in both cell lines. Indeed, cell shrinkage (Figure 3, arrow), pyknosis, and chromatin condensation (Figure 3, arrowhead), cell detachment from plate (Figure 3, circle) were detected after Giemsa staining. All cell death signs were reduced in FCoV-infected cells treated with the AhR inhibitor (Figure 3).

After AO/PI staining, CRFK and A72 cells were analyzed by a fluorescence microscopy to detect both viable and dead cells. In both cell lines, pretreatment with CH223191 resulted in an increase in green fluorescence (AO) (Figure 3b,f), and in a decrease in PI fluorescence of infected cells compared to FCoV untreated groups (Figure 3b,f). These results, confirmed by integrated density quantification (Figure 3c,d,g,h), showed that CH223191 determined a strong protection of CRFK and A72 cells during FCoV infection.

### 3.3. AhR Inhibitor Decreases Virus Yield During FCoV Infection: Standard Curve and Virus Quantification in CRFK and A72 Cells

**Figure 4:**
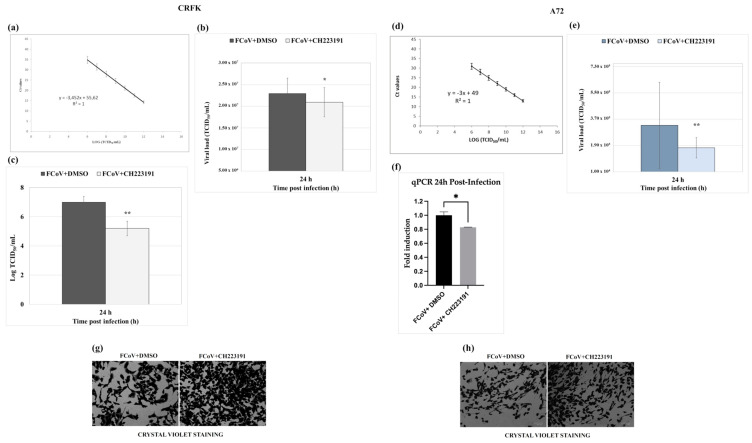
The AhR inhibitor CH223191 decreases FCoV viral load during infection in CRFK and A72 cells. Cells pretreated or not pretreated with the AhR inhibitor CH223191 and infected with FCoV. (**a**,**b**) In CRFK cells, at 24 h p.i., viral load (TCID_50_/mL) was evaluated by RT-qPCR, as described in the Materials and Methods section. Significant differences between FCoV-infected cells and AhR-inhibitor-treated infected cells are indicated by probability *p*. * *p* < 0.05. (**c**) At 24 h p.i., in CRFK cells, virus yield was evaluated by TCID_50_ method and reported as Log TCID_50_/mL. Significant differences between FCoV-infected cells and AhR-inhibitor-treated infected cells are indicated by probability *p*. ** *p* < 0.01. (**d**,**e**) In A72 cells, at 24 h p.i., viral load (TCID_50_/mL) was evaluated by RT-qPCR by the means of a standard curve. Significant differences between FCoV-infected cells and AhR-inhibitor-treated infected cells are indicated by probability *p*. ** *p* < 0.01. (**f**) The fold induction of FCoV gene coding for NP in A72-infected cells. Significant differences between FCoV-infected cells and AhR-inhibitor-treated infected cells are indicated by probability *p*. * *p* < 0.05. (**g**,**h**) CPE in CRFK and A72 cells by crystal violet staining was evaluated by ZOE Cell Imager. Scale bar: 100 µm. Results of one experiment representative of three independent experiments were reported.

RT-qPCR was employed to determine the AhR inhibitor’s capacity to induce the decrease in virus yield. FCoV virus yield was significantly diminished by the AhR inhibitor CH223191 in CRFK cells (Figure 4a,b) and A72 cells (Figure 4d,e). Thus, the replication rate of FCoV in CRFK and A72 cells pretreated with CH223191 was significantly lower than in untreated ones.

### 3.4. AhR Inhibitor Decreases Viral NP Gene Expression in A72 Cells

To quantify the relative expression levels of the mRNA coding for the nucleocapsid protein NP in FCoV-infected cells in the presence and absence of the inhibitor CH223191, real-time PCR was performed. Although A72 cells permit low-efficiency FCoV replication [46], a reduction in NP gene expression in the presence of the inhibitor compared to the untreated infected cells was observed (Figure 4f). 

### 3.5. AhR Inhibitor Decreases Virus Yield During FCoV Infection: CPE Evaluation

Following FCoV infection in CRFK cells, a significant (*p* < 0.01) reduction in FCoV virus titer (expressed in Log) was observed at 24 h p.i. in CH223191 pretreated groups (Figure 4c) compared to untreated infected cells. 

Furthermore, during FCoV infection in CRFK at 24 h p.i., CPE was increased (Figure 4g). Indeed, the detection of morphological alterations, such as the development of typical syncytia in CRFK giant cells, accompanied by detachment from the culture plate in FCoV-infected groups [55], was observed (Figure 3a and Figure 4g). These signs were extremely reduced by the presence of AhR inhibitor (Figure 3a and Figure 4g). Similar results were also obtained following FCoV infection in A72 cells (Figure 3e and Figure 4h).

Overall, the different methods used to assess virus yield in CRFK, as well as in A72 cells, all demonstrated that the AhR inhibitor CH223191 significantly reduced FCoV yield during infection. In addition, the inhibitor also caused a reduction in NP gene expression during FCoV infection in A72 cells.

### 3.6. AhR Was Expressed in CRFK Cells

**Figure 5:**
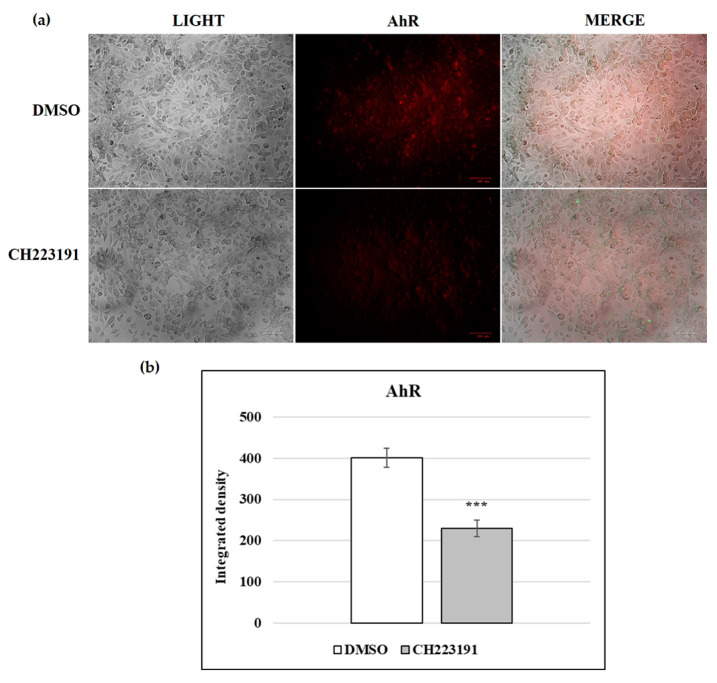
AhR expression in CFRK cells. (**a**) AhR inhibitor CH223191 significantly decreased the expression of AhR. Scale bar: 100 µm. (**b**) Bars represent the mean ratio generated from the integrated density (product of the area and mean intensity of fluorescence) of the AhR expression evaluated by ImageJ. Error bars represent standard deviation measurement. Significant differences between DMSO and AhR-inhibitor-pretreated cells are indicated by probability *p*. *** *p* < 0.001. The results of one experiment representative of three independent experiments were reported.

Immunofluorescence staining was employed to determine the expression of AhR in CFRK cells and A72 [30]. Figure 5 shows the expression of AhR in CFRK cells and how AhR expression was significantly inhibited by the CH223191 inhibitor. 

### 3.7. FCoV Infection Activates the Expression of AhR 

**Figure 6:**
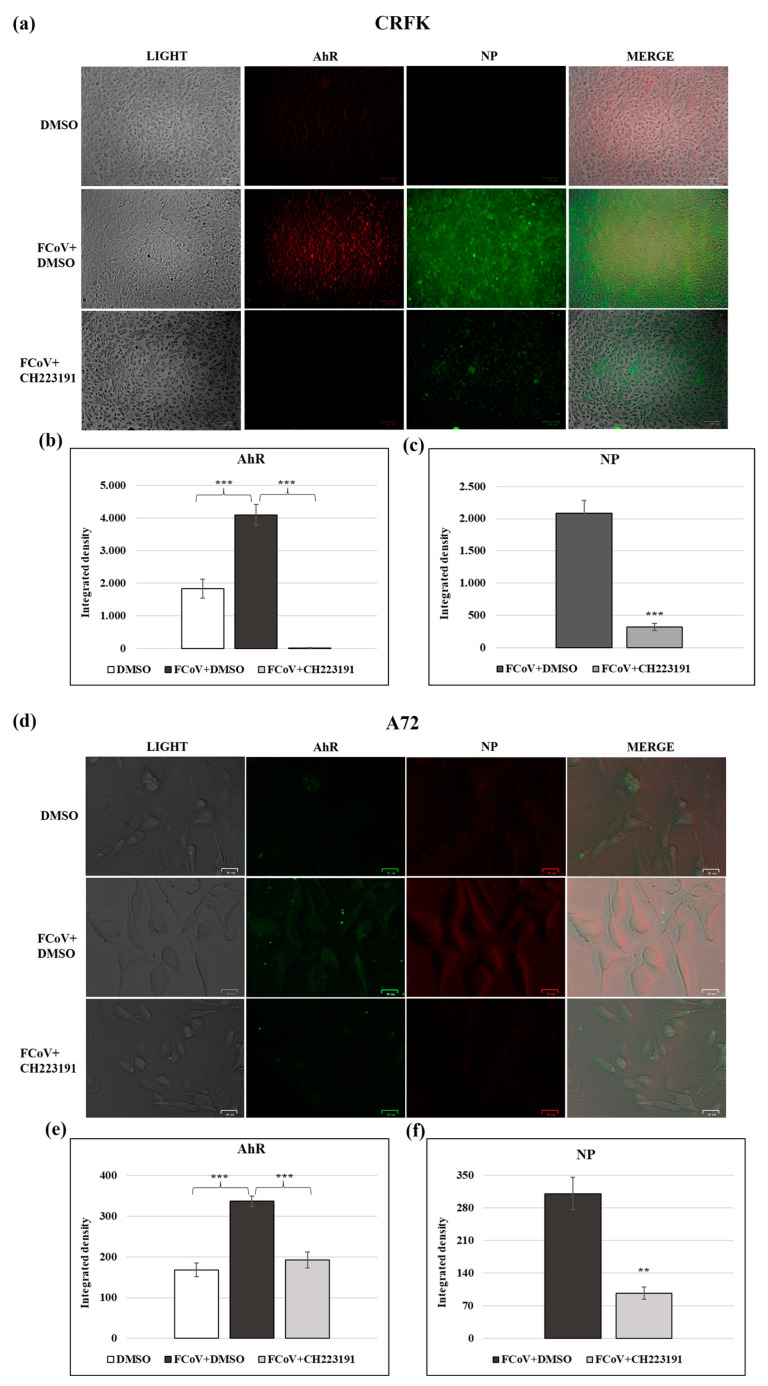
FCoV infection activates the expression of AhR and the AhR inhibitor downregulates AhR and NP during FCoV infection in CRFK and A72 cells. Cells were infected with FCoV for 24 h, and, using secondary antibodies in different colors following the expression of AhR and NP proteins s in CRFK and A72 cells, immunofluorescence staining was performed. (**a**,**d**) In both FCoV-infected cell lines, immunofluorescence staining indicated a significant upregulation of AhR and NP expressions. Following infection, the pretreatment with the AhR inhibitor CH223191 induced a downregulation of both AhR and NP expressions. Scale bar: 25 and 100 µm. (**b**,**e**) Bars are the mean ratio generated from the integrated density (product of the area and mean intensity of fluorescence) of the AhR expression during FCoV infection. Significant differences between FCoV-infected cells and AhR-inhibitor-treated infected cells are indicated by probability *p*. *** *p* < 0.001. (**c**,**f**) The integrated density graph of the NP expression during FCoV infection. Significant differences between FCoV-infected cells and AhR-inhibitor-treated infected cells are indicated by probability *p*. *** *p* < 0.001 in CRFK cells, and ** *p* < 0.01 and *** *p* < 0.001 in A72 cells. The integrated density was calculated by ImageJ. Error bars represent standard deviation measurement. The results of one experiment representative of three independent experiments were reported.

The AhR expression in CRFK and in A72 cells was significantly increased during the FCoV infection (Figure 6). These results were also confirmed by integrated density measurements indicating a 2.2- and 2.0-fold upregulation of AhR in infected CRFK and A72 cells, respectively, compared to the uninfected group (Figure 6a,d). A remarkable difference in x-axis (integrated density) limits was, however, observed (Figure 6b,e).

### 3.8. Inhibitor CH223191 Downregulates Both AhR and NPExpression During FCoV Infection

Following FCoV infection in CRFK and A72 cells, AhR and NP expressions were analyzed. NP was expressed during FCoV infection and the pretreatment of both cell lines with the AhR inhibitor CH223191 induced a downregulation in the expression of AhR and NP (Figure 6a,d). These data were confirmed by integrated density fluorescence measurement for both AhR (Figure 6b,e) and NP (Figure 6c,f).

These results indicate that FCoV infection activated the expression of AhR in both cell types. The AhR inhibitor downregulated AhR (Figure 6b–e) and NP (Figure 6c–f) (see differences in x-axis of integrated density limits).

### 3.9. FCoV Infection Activates the Expression of CYP1A1 

**Figure 7:**
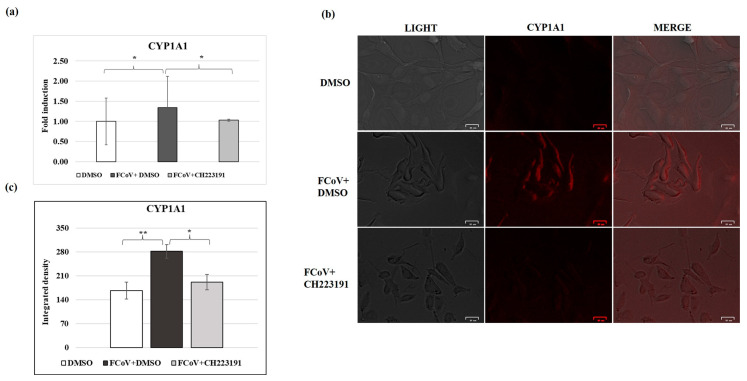
FCoV activates the expression of CYP1A1 (AhR signaling) during infection in A72 cells. Cells, pretreated or not pretreated with the AhR inhibitor, were infected with FCoV for 24 h. Then, (**a**) RNA was collected and quantified for mRNA levels of CYP1A1 by qRT-PCR. The data display the results of one experiment representative of three independent experiments; (**b**) immunofluorescence staining with antibody which recognizes CYP1A1 was performed. Scale bar: 25 µm. (**c**) Bars are the mean ratio generated from the integrated density (product of the area and mean intensity of fluorescence) of the CYP1A1 expression during FCoV infection. The integrated density was measured by ImageJ. Error bars represent standard deviation measurement. Significant differences between DMSO- and FCoV-infected cells, as well as between FCoV-infected cells and AhR-inhibitor-treated infected cells for CYP1A1 gene and protein expression are indicated by probability *p*. * *p* < 0.05 and ** *p* < 0.01. The results of one experiment representative of three independent experiments were reported.

The expression of AhR signaling, by assessing cytochrome CYP1A1, was evaluated in A72 cells during FCoV infection (Figure 7). Using qPCR and immunofluorescence, a significant increase in cytochrome expression was observed in infected cell groups, while the pretreatment with CH223191 led to a marked downregulation of gene (Figure 7a) and protein CYP1A1 expression in A72-infected cells (Figure 7b,c).

### 3.10. Lysosomes Are Involved in FCoV Infection

**Figure 8:**
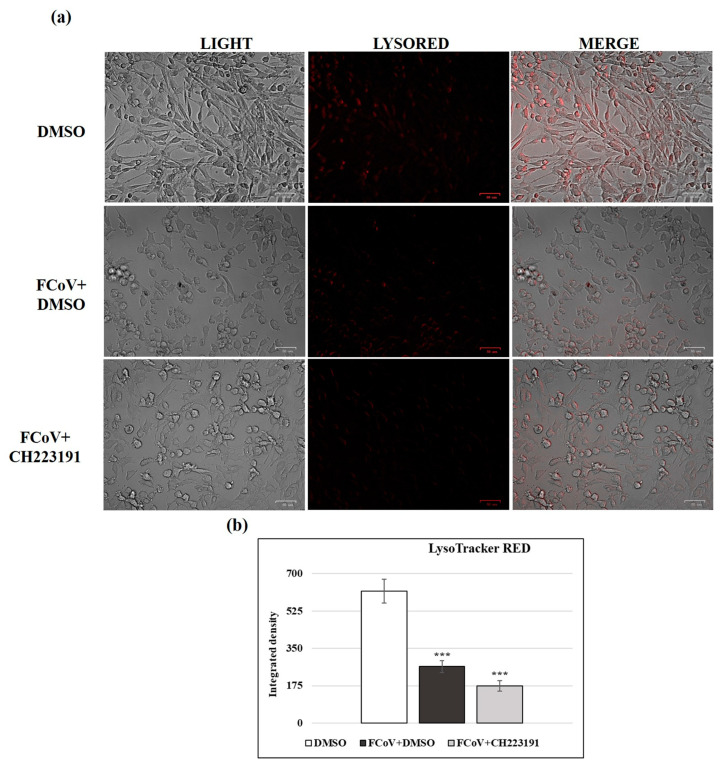
FCoV deacidifies lysosomes during infection in CRFK cells and the AhR inhibitor CH223191 further deacidifies them. (**a**) LysoRed staining of FCoV-infected cells compared to FCoV-infected cells pretreated with CH223191. Scale bar: 50 µm. (**b**) Bars indicate the mean ratio obtained by the integrated density of LysoTracker measured by ImageJ. Error bars indicate standard deviation quantification and significant differences are denoted by probability *p*. *** *p* < 0. 001.The results of one experiment representative of three independent experiments were reported.

Lysosomes, cellular organelles, are characterized by acidity induced by a proton pump. Following FCoV infection, lysosomes were treated by LysoRed staining, which usually marks lysosomes in live cells. In CRFK control cells (DMSO), a low pH structure was observed, while deacidification was detected after the pretreatment with CH223191 (Figure S1). FCoV infection in CRFK cells was responsible for cellular deacidification (Figure 8a,b), which was further alkalinized by the AhR inhibitor (Figure 8a,b).

## 4. Discussion

Although FIP is a lethal disease in felines, therapeutic strategies are currently limited and highly toxic. To date, based on the different mechanisms of action involved in the FCoV life cycle, several therapeutic approaches were evaluated [10,61,62]. Compounds acting by inhibiting cell to cell fusion, endocytosis, translation, host proteins and protease, have been tested. For example, hydroxychloroquine, a drug approved for the treatment of malaria and immune-mediated diseases in humans, associated with interferon-ω, is effective against FIPV in vitro [63]. Itraconazole, an antifungal [64]; sinefungin, a nucleoside antibiotic [65], 5-amino levulinic acid [66], and doxycycline [67] inhibit FCoV infection. ERDRP-0519, a non-nucleoside inhibitor, targeting viral RNA polymerase, is highly effective against FIPV [68]. A recent alternative to euthanasia, which often makes FIP curable, is due to the treatments with nucleoside analogs such as remdesivir [69] and GS-441524, a derivate of remdesivir [10,61,70,71,72,73,74,75,76,77]. Indeed, they are effective against FIP both in the short and long term [78], although their use may induce toxic effects, such as acute progressive azotemia and multifocal urolithiasis [79]. Promising results were found with a new therapy based on unlicensed molnupiravir [80,81]. 

The involvement of AhR during CoVs infection was recently reported [22,27,28,29,30,31,32,34,35,37]. In several mammalian cells, a different expression of AhR was observed [22]. In this study, we found, for the first time, that AhR was expressed in CRFK cells, as well as in A72 cells [30], and that it was significantly activated during FCoV infection [27,28,29,30,31,32]. In addition, the involvement of AhR signaling was explored, and both the gene and protein levels of CYP1A1, a downstream target protein of AhR, were upregulated during FCoV infection, similarly to other CoVs infections [29,31,32,82]. In contrast, treatment with AhR antagonists, such as CH223191, reduced the expressions of AhR and CYP1A1. Similarly, via AhR, the inhibitor induces a suppression of MHV, SARS-CoV-1, MERS-CoV, SARS-CoV-2, HCoV-229E, and CCoV infection in mammalian cells [28,29,30]. 

As previously observed in A72 cells [30] and confirmed in this work, no toxic effects in CRFK and A72 cells were observed using CH223191 at a dose of 2 µm, a concentration that significantly increased cell viability during FCoV infection. In addition, the AhR inhibitor reduced signs of morphological cell death related to FCoV infection in CRFK [54] and in A72 cell cultures (as shown in this study). Herein, a significant reduction in viral titers was observed in both CRFK and A72 cell lines, although A72 cells allowed for FECV (isolate “München”) replication with the appearance of evident cytopathic effects, in contrast to what was observed after infection with FCoV type I, strain C3663, infection which efficiently induces FIP [45].

These results suggest that FCoV is also responsible for the upregulation of AhR which is commonly used by CoVs to promote their replication [28,29,30]. Interestingly, a novel form of the co-expression of NP and AhR was observed in FCoV-infected CRFK cells (Figure 7), although further data are required to confirm these findings. 

Following FCoV infection, the pretreatment of CRFK and A72 cells with CH22319, an AhR inhibitor, resulted in the downregulation of AhR expression, as well as NP, the viral nucleoprotein that exhibits higher stability than the spike protein [83,84,85]. To develop potential inhibitors against CoVs, NP has a crucial role in host–pathogen dynamics, being involved in viral genome binding [86]. 

CoVs such as SARS-CoV-1, MERS-CoV, MHV, and SARS-CoV-2 enter cells by endocytosis. Burkard et al. [87] demonstrated that the entry of FCoV into the host cells also depends on trafficking to lysosomes. Herein, following FCoV infection in CRFK cells, using the biotype FECV (isolate “München”), the deacidification of lysosomes, which is correlated with a reduction in virus yield, was induced by the AhR inhibitor. Hence, we hypothesize that such mechanisms may render cell entry more difficult for the virus. Interestingly, Regan et al. [88] showed that the cell entry of FECV strain WSU 79-1683 (FECV-1683 depends on the low pH of endocytic compartments. On the other hand, the host cell entry of FIPV strains WSU 79-1146 (FIPV-1146) and FIPV-DF2 occur independently of low pH [88]. Apart from that, the general mechanism of lysosomal deacidification during CoVs infection is not fully elucidated yet. This could be the result of indirect effects, including excessive cargo (due to viruses) and/or distresses in proton pumps or in ion channel trafficking [38]. Interestingly, it was recently found that the lysosome–nucleus signaling pathway involves AhR that senses lysosomal cystine during ferroptosis in cancer cells [89], highlighting a novel correlation between lysosomes and AhR, which should be further investigated also during virus infections. 

Herein, to increase the knowledge of the mechanism of action in virus–host interaction, the implication of AhR in FCoV infection was found in vitro. Moreover, the involvement of CYP1A1, an identified effector downstream of AhR, was also found to be regulated by FCoV infection.

Although a unique member of signaling was assessed, the inhibition of AhR by CH223191 controlled FCoV replication, identifying AhR as a new target useful to identify antiviral drugs suitable to fight CoVs. Therefore, our results support the idea that the upregulation of AhR might be a common strategy used by CoVs to stimulate viral replication. Additionally, we hypothesize that the host response to CoVs infection may be controlled by AhR ligands. Further studies could define the current study on the role of AhR signaling in FCoV infection. However, these results highlight the importance of studying the mechanism of action in in vitro models, using animal CoVs to avoid handling highly dangerous human CoVs.

## Data Availability

The data that support the findings of this study are available from the corresponding authors upon reasonable request.

## Supplementary Materials

The following supporting information can be downloaded at: https://www.mdpi.com/article/10.3390/v17020227/s1, Original images used for the quantification of fluorescence in Figure 3, Figure 5, Figure 6, Figure 7 and Figure 8. Figure S1. The AhR inhibitor CH223191 deacidifies lysosomes in CRFK cells.
