# Supplementary material for: In Vitro Evaluation of Aryl Hydrocarbon Receptor Involvement in Feline Coronavirus Infection
Source: Viruses. 2025 Feb 6;17(2):227. doi: 10.3390/v17020227 (PMC11860311; doi:10.3390/v17020227)
Supplement: Supplementary file 1 [file viruses-17-00227-s001.zip › viruses-3377435-supplementary.pdf]

Supplementary information

In Vitro Evaluation of Aryl Hydrocarbon Receptor Involvement in Feline Coronavirus Infection

Luca Del Sorbo [1], Rosa Giugliano [1,2], Claudia Cerracchio [1], Valentina Iovane [3], Maria Michela Salvatore [4,*], Francesco Serra [5], Maria Grazia Amoroso [5], Francesco Pellegrini [6], Martina Levante [5], Paolo Capozza [6], Georgia Diakoudi [6], Massimiliano Galdiero [2], Giovanna Fusco [5], Annamaria Pratelli [6,*], Anna Andolfi [3,7] and Filomena Fiorito [1,7,*]

[1] Department of Veterinary Medicine and Animal Production, University of Naples Federico II, Naples, Italy
[2] Department of Experimental Medicine, University of Campania Luigi Vanvitelli, Naples, Italy
[3] Department of Agricultural Sciences, University of Naples Federico II, Portici, Naples, Italy
[4] Department of Chemical Sciences, University of Naples Federico II, Naples, Italy
[5] Istituto Zooprofilattico del Mezzogiorno, Portici, Naples, Italy
[6] Department of Veterinary Medicine, University of Bari, Valenzano (Bari), Italy
[7] BAT Center-Interuniversity Center for Studies on Bioinspired Agro-Environmental Technology, University of Naples Federico II, Portici, Italy

* Correspondence: filomena.fiorito@unina.it (F.F.); mariamichela.salvatore@unina.it (M.M.S.); annamaria.pratelli@uniba.it (A.P.)

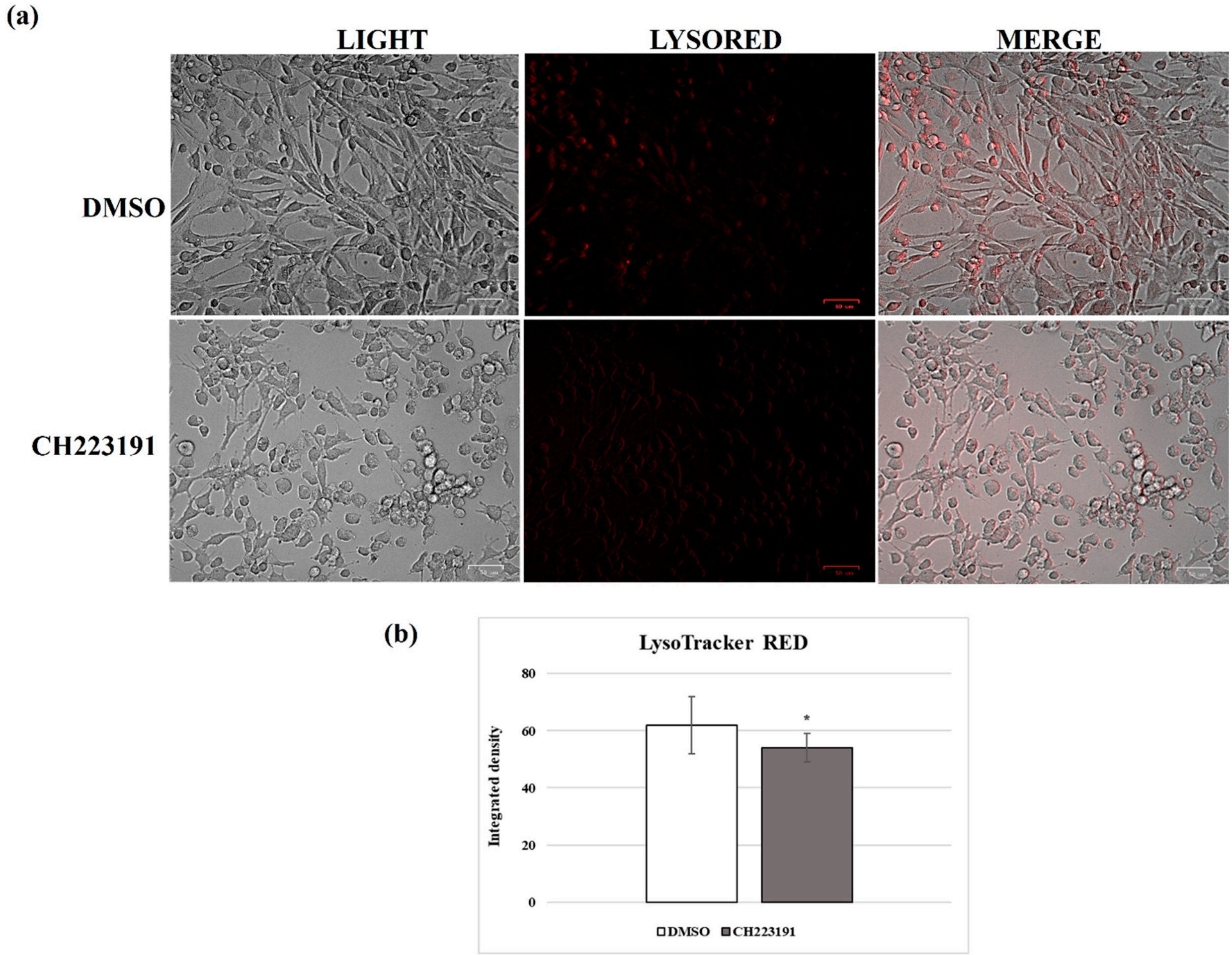

Supplementary Figure S1. The AhR inhibitor CH223191 deacidifies lysosomes in CRFK cells. (a) LysoRed staining of DMSO control group compared to cells pretreated with CH223191. Scale bar 50µm. (b) Bars indicate the mean ratio obtained by the integrated density of LysoTracker calculated by ImageJ. Error bars represent standard deviation quantification and significant differences are indicated by probability *p*. * $p<0.05$. The results of one experiment representative of three independent experiments were reported.
